# The pathogenesis of gut microbiota in hepatic encephalopathy by the gut–liver–brain axis

**DOI:** 10.1042/BSR20222524

**Published:** 2023-06-15

**Authors:** Ruirui Zhu, Liwen Liu, Guizhen Zhang, Jianxia Dong, Zhigang Ren, Zhiqin Li

**Affiliations:** 1 Department of Infectious Diseases, the First Affiliated Hospital of Zhengzhou University, Zhengzhou 450052, China; 2 Gene Hospital of Henan Province; Precision Medicine Center, the First Affiliated Hospital of Zhengzhou University, Zhengzhou 450052, China; 3 Jinan Microecological Biomedicine Shandong Laboratory, Jinan 250000, China

**Keywords:** Fecal microbiota transplantation, Gut microbiota, Gut-brain-liver axis, Hyperammonemia

## Abstract

Hepatic encephalopathy (HE) is a neurological disease occurring in patients with hepatic insufficiency and/or portal-systemic blood shunting based on cirrhosis. The pathogenesis is not completely clear till now, but it is believed that hyperammonemia is the core of HE. Hyperammonemia caused by increased sources of ammonia and decreased metabolism further causes mental problems through the gut–liver–brain axis. The vagal pathway also plays a bidirectional role in the axis. Intestinal microorganisms play an important role in the pathogenesis of HE through the gut–liver–brain axis. With the progression of cirrhosis to HE, intestinal microbial composition changes gradually. It shows the decrease of potential beneficial taxa and the overgrowth of potential pathogenic taxa. Changes in gut microbiota may lead to a variety of effects, such as reduced production of short-chain fatty acids (SCFAs), reduced production of bile acids, increased intestinal barrier permeability, and bacterial translocation. The treatment aim of HE is to decrease intestinal ammonia production and intestinal absorption of ammonia. Prebiotics, probiotics, antibiotics, and fecal microbiota transplantation (FMT) can be used to manipulate the gut microbiome to improve hyperammonemia and endotoxemia. Especially the application of FMT, it has become a new treated approach to target microbial composition and function. Therefore, restoring intestinal microbial homeostasis can improve the cognitive impairment of HE, which is a potential treatment method.

## Introduction

Hepatic encephalopathy (HE) is defined as brain dysfunction caused by liver failure and/or portal-systemic blood shunting based on cirrhosis [1], which is a serious complication of cirrhosis. Although the pathogenesis of HE has been incompletely understood, there are some hypotheses: toxicity of ammonia, manganese toxicity, neuronal cell death, systemic inflammatory response syndrome, and so on. Among them, the theory of ammonia poisoning has been studied the most, and hyperammonemia is considered as the core process of HE [2]. Recently, neural pathways have also been found to be involved in the mechanisms of cirrhosis and associated complications.

The gut contains more than 10 trillion microbes [3], which are involved in the formation of nervous, immune systems, or other basic processes during growth. With the progression of cirrhosis, harmful intestinal bacteria can secrete different metabolites to promote the occurrence of HE through the complex gut–liver–brain axis, resulting in central nervous system (CNS) dysfunction [4]. Therefore, intervening in the gut microbiome may be the treatment of choice at all stages of liver disease, as it reduces liver and nervous system exposure to intestinal toxins. The present review summarizes the changes in gut microbiota during the progression of liver cirrhosis to HE, how the gut microbiota affects this gut–liver–brain axis, and the therapeutic effect of HE by adjusting the gut microbiota.

## High ammonia metabolism and vagal mechanisms of HE

### Hyperammonemia

#### Sources of ammonia

Ammonia comes from the metabolism of food and tissue protein. After digestion in the small intestine, the food is broken down into amino acids. In intestinal mucosal cells, amino acids break down to produce ammonia. In the colon, the bacteria urease in the intestine can decompose urea to produce ammonia. The gut is the main site of ammonia production. In addition to the intestinal tract, the kidney can both secrete and excrete ammonia. Glutamine is hydrolyzed to glutamate and ammonia by glutaminase in proximal renal tubule cells. Approximately 70% of ammonia is reabsorbed into the circulation by renal veins [5], so the kidneys are also the source of ammonia. The remainder is excreted in the urine, thus regulating the body’s acid–base balance. Moreover, ammonia can be produced anywhere in the tissue, both by amino acid deamination and amine decomposition.

#### Ammonia metabolism

Under normal physiological circumstances, the vast majority of ammonia metabolism in the liver has two ways: one is the urea cycle, the other is to form glutamine for temporary detoxification [6]. A small amount of ammonia is excreted from the kidneys as ammonium salt in the urine.

Ammonia absorbed from the colon is delivered to the liver by the portal blood. Hepatocytes from near the portal vein convert ammonia to urea via the urea cycle. Urea is put into the blood and eventually excreted through the kidneys, with a small amount excreted through sweat.

Ammonia that has not gone through the urea cycle is absorbed and detoxified by hepatocytes who contain glutamine synthetase, which has a high affinity for ammonia. At the same time, hyperammonemia stimulates glutamine synthesis [7]. Glutamine synthetase is found in almost all tissues, such as skeletal muscle, brain, heart, and lung, and it can catalyze the conversion of ammonia and glutamate to glutamine. This way guarantees that under normal physiological conditions, the liver nearly metabolizes all the gut-derived ammonia. However, such a way only should be considered as a temporary removal of ammonia, because the glutamine is catabolized to glutamate and ammonia in the gut and the kidneys. Therefore, glutamine is a major nontoxic interorgan ammonia carrier [8].

The kidneys use glutaminase to break down glutamine transported from each tissue. A small amount of NH_3_ combines with H+ in urine to form NH_4_^+^, which is excreted in the form of ammonium salts. However, severe liver disease can aggravate renal excretion, resulting in a decrease in the excretion of ammonia and an increase in blood ammonia [8].

In the progress of liver diseases, there is decreased capacity for urea synthesis resulting in the reduction of ammonia removal by the liver. But the activity of glutaminase in the small intestine of cirrhotic patients is increased, and the catabolism of glutamine in the kidneys, which decomposes glutamine to produce ammonia, leading to the raising of blood ammonia levels [9,10]. Ammonia is particularly toxic to the brain; therefore, it has to be disposed of effectively.

#### Hyperammonemia metabolism

**Figure 1:**
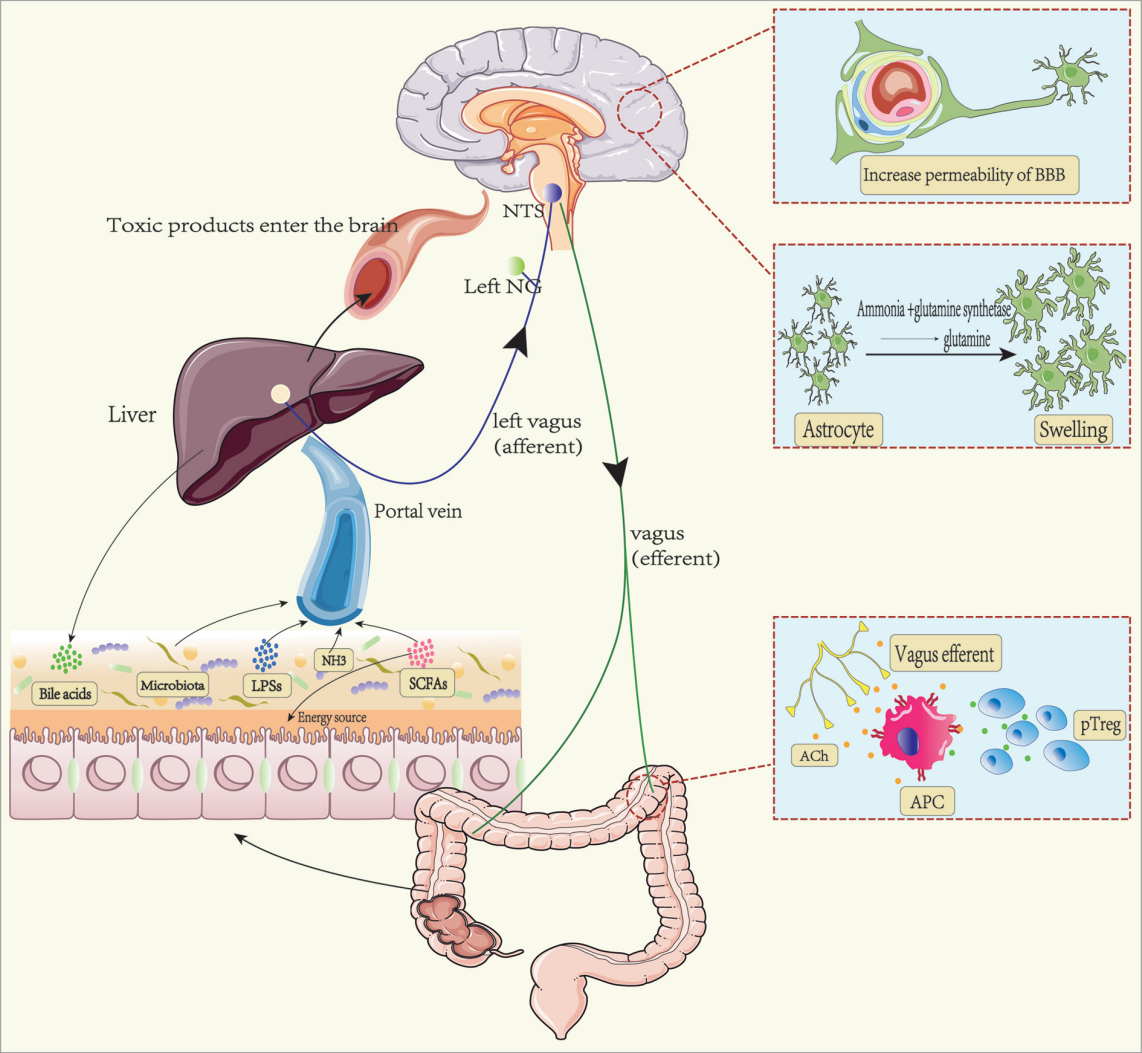
Ammonia and vagus nerve affect the body through the gut–liver–brain axis Ammonia and other products from the gut are delivered to the liver by the portal vein. When the liver is in a damaged state or portal-systemic shunting based on cirrhosis, unmetabolized ammonia and other toxic products can enter the brain through the blood–brain barrier (BBB). Under the synergy of inflammation of the brain, ammonia can cause neurocognitive impairment by making astrocyte swelling, brain cells lack of energy, and other mechanisms. These products also become afferent signals that transmit the signals to the nucleus tractus solitarius via the left nodose ganglion (NG). Vagal efferent fibers regulate peripheral regulatory T (pTreg) cells by acting on antigen-presenting cells (APC).

The levels of normal blood ammonia are 10–50 µmol/l [11]. Liver insufficiency and/or portal-systemic blood shunting based on cirrhosis can cause ammonia concentration increase. In patients with liver disease, blood ammonia is elevated due to impaired liver function. The brain can induce neurotoxicity because it is particularly sensitive to ammonia toxicity [12]. Therefore, under the condition of high blood ammonia, HE with neurocognitive impairment and altered consciousness level can be caused (Figure 1).

Most of the ammonia in the brain comes from the blood, small amounts are from the cerebrospinal fluid, and the rest is produced by brain metabolism [13]. In hyperammonemia, ammonia can enter the brain freely, independent of the blood–brain barrier (BBB), and it can be exclusively removed by astrocytes via glutamine synthetase. Astrocytes are the most commonly affected in patients with HE owing to the exclusive localization of glutamine synthetase within the CNS to astrocytes [14]. Ammonia is converted into glutamine by glutamine synthetase in astrocytes. The massive accumulation of glutamine increases the osmotic pressure of astrocytes, leading to cell swelling. The nervous system is composed of neurons and glial cells; however, the direct harm of ammonia toxicity to neurons was not significant, indicating the neuroprotective role of astrocytes [15].

Ammonia can also interfere with the tricarboxylic acid cycle (TCA cycle), making brain cells have less energy supply. Because ammonia can be an inhibitor of the TCA cycle, such as pyruvate dehydrogenase and α-ketoglutarate dehydrogenase, high ammonia concentrations interfere with the TCA cycle by inhibiting pyruvate dehydrogenase and α-ketoglutarate dehydrogenase. This explains the high levels of pyruvate and lactate in the brain of HE patients [16].

Studies are showing that high ammonia induces HE only when systemic inflammatory response syndrome is present. Because ammonia levels are poorly correlated with the severity of HE [17]. Hyperammonemia can induce neutrophil dysfunction and release reactive oxygen species, triggering systemic oxidative stress and inflammation, which exacerbate the harmful effects of hyperammonemia on the brain [18]. Hyperammonemia can activate microglia that causes neuroinflammation by releasing inflammatory factors. This neuroinflammation mediates the deleterious effects of hyperammonemia on motor and cognitive function [19]. Astrocyte swelling caused by hyperammonemia may be a trigger for inflammation in the brain. Astrocytes, as components of the BBB, may be the key cells between ammonia and inflammation in the brain [20].

### Vagal mechanisms

The gastrointestinal tract is highly innervated, while gut microbiota can also regulate the nervous system. Neurological differences were found in comparison with conventional mice and germ-free (GF) mice [21]. The gut microbiota modulates the CNS via the enteric nervous system (ENS). Colonizing the intestinal microbiota of conventionally raised mice in GF mice can alter the anatomical structure of the ENS and intestinal transit function in a 5-HT-dependent manner [22]. The interactions of the gut–liver–brain axis are not only body fluids but also neural connections, especially the vagus nerve (VN).

Neural pathways are involved in immune regulation and inflammation control at multiple sites in the body. The VN also regulates immunity and inflammation in the liver and gastrointestinal tract. It has been reported 20 years ago that lipopolysaccharides (LPSs)-induced tumor necrosis factor (TNF) production in the liver can be attenuated after stimulation of the cervical VN. This is due to the release of acetylcholine (ACh) from efferent VN by acting on α-7-nicotinic ACh to inhibit TNF production by macrophages [23]. Hepatic vagotomy performed on diet-induced nonalcoholic steatohepatitis mice significantly increased the levels of TNF, IL-12, thereby exacerbating liver inflammation [24]. On the other hand, by performing surgical interruption of the VN in mice with inflammatory bowel disease, a significant increase in colonic levels of TNF and other proinflammatory cytokines was found and exacerbated the severity of acute colitis. By contrast, vagotomy has no effect on disease severity in macrophage-colony stimulating factor-deficient mice, supporting macrophage-mediated regulation [25].

The VN, which travels from the brainstem to the abdomen as the longest nerve of the human body, innervates most organs, especially in the gastrointestinal tract. The VN is a key component of the autonomic nervous system. The anti-inflammatory effect of the VN on the gastrointestinal tract is mediated through the cholinergic anti-inflammatory pathway [26]. It refers to the action of vagal efferent fibers on enteric neurons that release ACh. ACh can bind to α-7-nicotinic ACh receptors of those macrophages to inhibit the release of TNF-α, a proinflammatory cytokine [27].

The hepatic branch of the VN plays an important role in transmitting information from the liver to the brain. In the gut–liver–brain axis, nutrients, bacterial products, and toxins from the intestine that are transported to the liver through the portal vein become afferent signals to the hepatic VN, transmit the signals to the nucleus tractus solitarius of the brainstem via the left nodose ganglion (NG). Vagal efferent fibers release ACh acting on the muscarinic acetylcholine receptors (mAChR) of intestinal antigen-presenting cells (APC). Activation of mAChR in human APC directly induces aldehyde dehydrogenase (ALDH) gene expression, regulates colonic peripheral regulatory T (pTreg) cells expression by regulating ALDH expression and retinoic acid synthesis in APC, thereby regulating intestinal immunity [28,29]. This completes the gut–liver–brain–gut signaling (Figure 1). Subdiaphragmatic vagotomy (VGx) in normal mice decreases the abundance of colonic pTreg cells. Dysfunction of this liver–brain–gut neural arc predisposes the gut to inflammation. It was found that patients with a history of depression were more likely to have inflammatory bowel disease and that antidepressant treatment had a selective protective effect on inflammatory bowel disease [30]. This also validates that CNS can regulate the intestinal immune response.

## Gut microbiota characteristics of cirrhosis

A large number of microorganisms colonize the human gut and maintain a stable symbiotic or reciprocal relationship with the healthy hosts. The gut microbiota of healthy hosts is in a balanced state in the distribution of bacteria. Meanwhile, the intestinal flora also has the ability to synthesize essential vitamins and amino acids, and promote the absorption of mineral elements, so it plays a key role in maintaining the stability of the intestinal microecosystem [31]. It has been found that the change in gut microbiota is associated with many diseases, and it is also accompanied by the change of gut microbiota in the occurrence and development of cirrhosis and its complications.

Cirrhosis is the common end-stage of various chronic liver diseases. The reasons for cirrhosis are more, such as viral hepatitis, alcoholic liver disease, nonalcoholic fatty liver disease, and other metabolic or autoimmune disorders [32]. Viral hepatitis and alcohol abuse are the most common causes of liver cirrhosis. In China, hepatitis B virus infection is the major cause. For another, alcohol and hepatitis C virus infection are the common causes in Western countries [33].

The gut microbiota in cirrhosis is mainly characterized by low microbial diversity, the decrease of potential beneficial taxa, and the overgrowth of potential pathogenic taxa [34]. The findings of Chen et al. showed that microbial diversity had a tendency to be reduced according to the Shannon diversity index in the cirrhosis group, and the intestinal microbial community composition changed both in terms of phyla and families. At the phylum level, bacteroidetes was decreased, whereas proteobacteria and fusobacteria were enrichment in the cirrhosis group compared with healthy controls. At the family level, enterobacteriaceae, pasteurellaceae, streptococcaceae, and veillonellaceae were increased significantly in patients with cirrhosis. Lachnospiraceae was decline [35,36]. They also found that patients with cirrhosis of different etiologies (HBV-related and alcohol-related) had similar fecal microbial communities. There was no statistical difference at the phylum level between them, but at the family level, prevotellaceae was riched in alcoholic cirrhosis patients compared with HBV-related cirrhosis patients (Table 1). This led to the conclusion that although microbial diversity and individual differences in cirrhosis were great, the intestinal microbiome was significantly affected by cirrhosis at the family level. Another study of intestinal microbiota in cirrhotic patients with hepatitis B virus infection showed a lower count of firmicutes, especially in *F. prausnitzii*, clostridium clusters XI and XIVab, bifidobacterium [37] (Table 1). Bajaj et al. further described the significant differences in fecal microbial composition between cirrhosis and healthy controls (Table 1). The abundance of lachnospiraceae, ruminococcaceae, and clostridium incertaesedis XIV was significantly higher in the control group; however, lactobacillaceae, alcaligenaceae, fusobacteriaceae, enterobacteriaceae, and leuconostocaceae were significantly lower in the controls compared with cirrhotic patients [34]. In addition to significant differences in the fecal microbiome between healthy individuals and cirrhotic patients, they also found differences between the colonic mucosal microbiome and fecal microbiome in cirrhotic patients, and these differences persisted in the HE group [38]. They also first investigated and found relative stability of the microbiota over time within cirrhotic patients whose disease remained unchanged. This could help use the microbiome as a potential biomarker [39].

**Table 1:** Fecal microbial flora in cirrhosis

Groups	Lower microbiome	Higher microbiome
Cirrhosis [35,36]	Bacteroidetes	Proteobacteria^*^
	Lachnospiraceae	Fusobacteria
		Enterobacteriaceae^**^
		Pasteurellaceae^***^
		Streptococcaceae^****^
		Veillonellaceae^*****^
Healthy controls [34]	Enterobacteriaceae	Lachnospiraceae^******^
	Fusobacteriaceae	Ruminococceae^******^
	Alcaligenaceae	Clostridium incertaesedis XIV
	Lactobacillaceae	
	Leuconostocaceae	
HBV-related cirrhosis [35–37]	Firmicutes	
	F. prausnitzii	
	Clostridium clusters XI and XIVab	
	Bifidobacterium	
Alcohol-related cirrhosis [35–37]		Prevotellaceae

This table contains two groups of fecal microbial differences. One is a comparison of fecal microflora between cirrhotic patients and healthy controls. In patients with cirrhosis, there is a significant increase in pathogenic flora, such as Proteobacteria, Fusobacteria, Enterobacteriaceae, Pasteurellaceae, Streptococcaceae, Veillonellaceae. These pathogenic flora are associated with inflammation, liver fibrosis, hepatic steatosis, and central nervous. The other is a comparison between patients with cirrhosis due to different etiologies.

**Effects of bacteria on diseases:**

* An enhanced Proteobacteria proportion could be the most prominent trigger of hepatic steatosis by disruption of the gut–liver axis [90].

** Enterobacteriaceae were found to be positively correlated with astrocyte swelling.

*** The relative abundance of Pasteurellaceae was independent factors predicting mortality rate [91].

**** A positive correlation was observed between Child-Turcotte-Pugh score and Streptococcaceae [92].

***** Veillonellaceae may be positively correlated with the severity of liver fibrosis [93].

****** Lachnospiraceae and Ruminococceae as beneficial bacterias produce short-chain fatty acids (SCFAs) and improve the intestinal barrier. They also have a robust correlation with inflammatory cytokines (interleukin [IL]-6, TNF-α, IL-2) [48,91].

## Gut microbiota characteristics of HE

Decompensated cirrhosis is characterized by the onset of several complications like HE and spontaneous bacterial peritonitis. There is a lot of data suggesting that HE is associated with dysbiosis of gut microbiota. According to the severity of manifestations, HE has been divided into a fully symptomatic overt HE (OHE) and a covert HE (CHE). CHE can be subdivided into an entirely asymptomatic condition, called minimal HE (MHE) [40]. Compared with cirrhotic patients without cognitive dysfunction, patients with both MHE and OHE had specific alterations of gut microbiota profile [41].

Bajaj et al. studied the fecal microbes in cirrhotic patients with and without HE. They found that the HE group had a higher abundance of veillonellaceae compared with the no-HE group. There were no obvious differences in other fecal microbiomes [42]. Whereas in the colonic mucosal microbiome, there is a significant difference between the HE and no-HE patients. Firmicutes such as members of genera veillonella, megasphaera, bifidobacterium, burkholderia, and enterococcus were higher in HE, whereas roseburia was higher in the no-HE groups [38] (Table 2).

**Table 2:** Higher microbiome in HE/no-HE

Groups	Fecal microbial flora	Mucosal microbiota
HE [38]	Veillonellaceae	Firmicutes
		Veillonella
		Megasphaera
		Bifidobacterium
		Burkholderia
		Enterococcus
no-HE [38]		Roseburia

This table lists the higher microbiome in the different groups. The colonic mucosal microbiota is different from the fecal microbiota between HE and no-HE groups. Altered colonic mucosal microbiota in HE patients is associated with poor cognitive performance and inflammation.

Altered colonic mucosal microbiota in HE patients is associated with poor cognitive performance and inflammation [41].

A study carried out a quantitative bacteriological analysis of fecal samples from outpatients with minimal HE showed that cirrhotic patients with MHE had a significant fecal overgrowth of *E. coli* and Staphylococcus [43]. In another study, streptococcus and veillonellaceae numbers were all higher in cirrhosis with and without MHE [44]. Studies on the linkage between the specific gut microbiota and cirrhosis-related brain dysfunction showed that cirrhotic patients with HE had a higher abundance pattern of staphylococcaceae, enterococcaceae, porphyromonadaceae, and lactobacillaceae compared with healthy controls and cirrhosis without HE [45]. These show that the gut microbiota will be altered as the disease progresses.

## Intestinal microecology promotes the occurrence and development of HE through the gut–liver–brain axis

HE is a complication of decompensated cirrhosis, presenting with cognitive and motor dysfunction. Changes in gut microbiota are aggravated with the progression of cirrhosis. The intestinal flora of HE patients decreased beneficial bacteria while increased potential pathogenic bacteria, which lead to a tremendous change in intestinal homeostasis (Figure 1). Changes in microbiome composition and metabolism can affect the human body along the gut–liver–brain axis.

Imbalance of gut microbiota can affect the synthesis of substances and release a large number of metabolites. The decrease of intestinal beneficial bacteria (such as lachnospiraceaea and ruminococcaeaea) resulted in the decrease of short-chain fatty acids (SCFAs) production [46], and the abnormal production of butyrate was particularly serious [47]. SCFAs are produced in the colon after the fermentation of carbohydrates by intestinal microorganisms, which can reduce intestinal inflammation, provide energy for colon epithelial cells, regulate colon pH, and improve intestinal barrier function [48]. Therefore, its reduction may raise pH and allow urease-producing bacteria to continue to grow and produce ammonia and endotoxin [49], leading to hyperammonemia, systemic inflammation, and impaired intestinal barrier. The permeability of the BBB was decreased when *Clostridium tyrobutyricum*, a butyrate-producing bacteria, was transplanted to GF mice [50]. Thus, butyrate not only maintains the integrity of the intestinal barrier, and reduces bacterial translocation but also has the ability to cross the BBB and promote hippocampal-dependent learning [51]. Therefore, the reduction of beneficial flora will lead to the dysfunction of the body.

The increase of potential pathogenic bacteria in the intestine leads to the rising of bacterial metabolites. Gut microbiota and its components, such as LPS, peptidoglycan, and bacterial lipoprotein, are released into the bloodstream. Elevated blood ammonia level in liver disease is a typical example. In addition to the increased ammonia production in the gut, the damaged liver is unable to effectively remove excess ammonia, thus leading to hyperammonemia. Moreover, LPS is the main component of gram-negative bacteria in the intestine, such as the overgrowth of enterobacteriaceae and streptococcaceae [44]. LPS is the main component of triggering systemic inflammation. Large amounts of LPSs entering the systemic circulation can lead to endotoxemia, increase BBB permeability and neuroinflammation, thus promoting the occurrence of HE [52]. Toxic metabolites can also directly lead to liver cell death and liver function deterioration.

The liver is bidirectional to the intestine via portal circulation and bile secretion, which is called gut–liver axis. Gut microbiota also affects bile acid production. In cirrhosis, there is less primary bile acid entering the intestine as a result of liver damage, and less secondary bile acid is produced by bacteria. These results cannot effectively inhibit the overgrowth of bacteria [53]. The study found that plasma bile acid levels are significantly increased in cirrhotic patients [54]. Consistently, the activated apical sodium-dependent bile acid transporter was found to promote intestinal bile acid reabsorption, thus increasing serum bile acid levels in the HE mouse model. Elevated serum deoxycholic acid (DCA) and chenodeoxycholic acid (CDCA) can activate Rac1 in vascular endothelial cells, resulting in an increase in permeability of the BBB [54]. Bile acids can allow it and other molecules to enter the brain, thus increasing the levels of bile acids in the cerebrospinal fluid [55]. The gut-dominant bile acid receptors have also been found in the nervous system, thus bile acids also can affect the CNS. The alteration in bile acid levels has been found in different neurological diseases, such as Alzheimer’s disease, Parkinson’s disease [56,57]. Bile acids in the gut also can affect the CNS indirectly through the gut–brain axis [58]. These may be related to neurotoxicity [59].

These various reasons eventually lead to intestinal inflammation, the increase of intestinal metabolites, and intestinal barrier permeability, which cause bacterial translocation and absorption of bacterial metabolites into the blood [60].

Factors entering the systemic circulation, such as ammonia, other bacterial products, and inflammation, can increase the permeability of the BBB, which eventually enters the brain [61]. The gut–brain axis is a bidirectional connection between the gut and the CNS. They can change information through the nervous system, endocrine system, and immune system [62]. To maintain a healthy gut–liver–brain axis, the body has three safeguards: a barrier of the intestinal epithelium, normal liver function, and the BBB. As an important barrier interface in the gut–brain axis, BBB plays a crucial role in stabilizing the CNS microenvironment [63]. Substances entering the brain can affect the structure and function of the CNS by directly affecting the function of glial cells or indirectly affecting the VN in the intestinal nervous system [64]. Moreover, systemic inflammation and hyperammonemia synergistically also enhance neurotoxicity in the central system.

In addition, specific gut microbes correspond to the damage in different structures of the CNS in the HE patients. They are ultimately associated with neuronal and astrocyte dysfunction in patients with cirrhosis. Beneficial bacteria (lachnospiraceae, ruminococcaceae, clostridium XIV) were negatively correlated with brain glial magnetic resonance spectroscopy expression of ammonia (higher glutamine + glutamate, lower myoinositol and choline). In contrast, the increase of potential pathogenic bacteria groups in cirrhosis and HE (streptococcaceae, enterobacteriaceae, lactobacillaceae, and peptostreptococcaceae) were positively correlated with ammonia and brain magnetic resonance spectroscopy manifestations. Enterobacteriaceae were found to be positively correlated with astrocyte swelling. Porphyromonadaceae is associated with neuronal damage [45].

Most importantly, in the case of cirrhosis, the hepatic VN may be a key link in the gut–liver–brain axis [65]. Compared with conventional cirrhotic mice, cirrhotic mice with hepatic vagotomy have lower intestinal dysbiosis and higher relative abundance of beneficial native taxa, such as Lachnospiraceaeae and Ruminococcaceae. This phenomenon shows that vagal output from the liver enhances the overgrowth of potential pathogenic bacteria. On the other hand, its steatosis was increased. The key genes related to hepatic lipid metabolism were altered, such as lipoprotein lipase was up-regulated and carboxylesterase, Ces1g and Ces2 were down-regulated [66,67]. Microglia and glial activation in the brain are usually associated with cirrhosis-related neuroinflammation. The study found that glial and microglia marker levels were higher in unvagotomized cirrhotic mice, while brain-derived neurotrophic factor (BDNF), which has anti-inflammatory effects, was higher in the brains of cirrhotic mice with hepatic vagotomy [68]. This suggests that neuroinflammation in cirrhosis may be related to factors other than glial cell activation. These data that vagal innervation of the liver plays an important role in the gut–liver–brain axis, which has implications for the pathogenesis of cirrhosis and associated complications.

## Fecal microbiota transplantation treats HE

**Figure 2:**
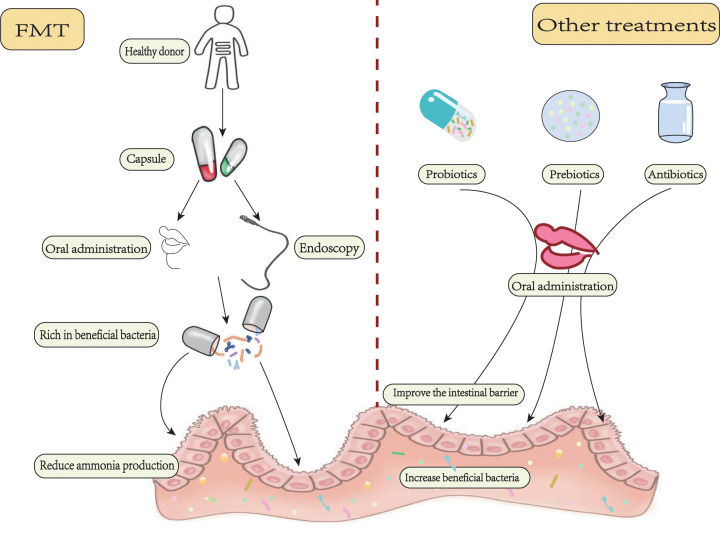
FMT and other methods treat HE FMT is the processing of feces from healthy donors into capsules that are transferred to patients by oral or endoscopic means. Select a single donor who is rich in beneficial flora for treatment. Beneficial bacteria that enter the gut can improve the recipient’s intestinal environment in a variety of ways. In addition, there are probiotics, prebiotics, antibiotics, and other programs. These methods can reduce bacterial urease activity and pH, improve the intestinal barrier, increase SCFAs production, enhance host immunity, and reduce ammonia production.

As gut microorganisms affect health and disease through the gut–liver–brain axis, microbial–host interactions play an important role in the pathogenesis of HE. Fecal microbiota transplantation (FMT) has become a new therapeutic approach to target microbial composition and function. The current treatments are targeting the gut microbiome, including diet, probiotics, prebiotics, and antibiotics. The standard care of HE, including rifaximin and lactulose, could improve the environment of the microbiota in the intestine. However, their therapeutic effect in practice is far from the ideal state, such as antibiotic resistance, and poor adherence due to gastrointestinal adverse effects of lactulose [69]. Therefore, the administration of specific microbial therapy has become a new approach. FMT involves transferring processed fecal materials from healthy donors to the patient to alter the composition of gut microbiome (Figure 2). By changing the intestinal flora, the effect of metabolites on the body may be fundamentally changed. It is an approved and effective treatment for other gastrointestinal diseases. This method has been used in ulcerative colitis and recurrent clostridium difficile infection [70,71]. It is also safe and effective to treat clostridium difficile infection in patients with cirrhosis [72].

The first attempt to use FMT to treat HE was reported by KAO et al. [73]. HE was treated in a 57-year-old man with grade 1–2 HE secondary to alcohol and hepatitis C cirrhosis. Cognitive assessment using the inhibitory controlled trial (ICT) and Stroop test showed that continuous FMT can improve the cognitive impairment of mild HE, and the beneficial effects of FMT were short-lived, requiring repeated treatment to maintain response. Bajaj et al. conducted a randomized controlled trial on the efficacy of FMT in HE and an experimental study on the long-term safety of FMT. In the randomized controlled trial, Bajaj et al. enrolled 20 cirrhotic patients with recurrent HE and randomly assigned them to the standard of care and the FMT group [74]. A single donor rich in beneficial bacteria was selected. The trial was followed up for 150 days. The experimental results showed that after FMT, the beneficial flora of the FMT group (lactobacillus, bifidobacteria, lachnospiraceae, and ruminococcaceae) was increased, and there were no FMT-related serious adverse events, including no bacterial infection, and FMT group showed significant improvement in cognition on day 20. The present study suggests that targeted therapy to supplement the defective microbiome can reduce HE recurrence.

A follow-up study of >12 months was conducted to show the long-term safety of FMT [75]. The result showed that hospitalization and HE episodes were apparently reduced in the FMT group compared with the control group, and FMT is considered to be safe for long-term use. After that, a phase 1 randomized placebo-controlled trial was studied to demonstrate the safety of capsular FMT. FMT capsules containing lachnospiraceae and ruminococcaceae were followed up for 5 months. Although one serious adverse event was reported in the FMT group, compared with 11 cases in the control group, FMT capsules could be tolerated and safe [76]. Mehta et al. in a retrospective study of ten patients with cirrhosis and recurrent HE who underwent FMT colonoscopy found that these patients had reduced Child-Pugh score, arterial ammonia concentration, and end-stage liver disease model score [77]. Therefore, FMT via colonoscopy is also an effective treatment option. Bajaj et al. evaluated the expression of antibiotic resistance genes (ARG) in decompensated cirrhotic patients before and after FMT by FMT capsules and enema FMT. Compared with baseline, β-lactamase expression was found to be reduced after FMT capsules. β-lactamase, vancomycin, and rifamycin ARG were significantly reduced at 4 weeks after FMT compared with placebo. In the enema FMT trial, β-lactamase and vancomycin ARG were reduced on day 7 after treatment compared with standard treatment. These data suggest that regardless of the route of administration, the ARG abundance of FMT is significantly decreased in decompensated cirrhosis, even in this advanced population [78]. In a study, two patients with cirrhosis secondary to hepatitis B had grade 2–3 HE recurrence after TIPS, so FMT was performed for three times. Donors with a high abundance of beneficial bacteria, such as ruminococcus, akkermansia, and oscillospiraceae, were selected and followed up for 1 year. After transplantation, the intestinal flora of the patients changed significantly, their condition also improved, the incidence of HE decreased, and Child-Pugh scores declined [79].

To study whether FMT could prevent the occurrence of HE, Wang et al. induced acute liver dysfunction rat model with carbon tetrachloride (CCl_4_). The rats then received FMT. The results described that FMT could improve HE grade, rat behaviors, and spatial learning capability. In addition, FMT prevented liver necrosis and gut mucosal barrier damage [80]. FMT is a promising new therapy, which can restore the diversity of intestinal flora in patients and achieve a therapeutic effect, but attention should be paid to preventing the transmission of pathogens through feces in donor screening.

## Probiotics, prebiotics, and antibiotics treat HE

The treatment aim of HE is to decrease intestinal ammonia production and intestinal absorption of ammonia, so whether FMT, probiotics, prebiotics, antibiotics or other treatments (Figure 2), all can reduce serum ammonia levels and intestinal-derived neurotoxic substances by accelerating intestinal transport and changing the metabolism and abundance of intestinal bacteria.

Probiotics are defined as living microbes that can bring health benefits when given in sufficient quantities to the host. By taking probiotics orally, the body can directly increase the number of good bacteria in the intestine. Probiotics entering the intestine can regulate the intestinal microbiota, reduce ammonia production by decreasing bacterial urease activity and pH, and protect the intestinal barrier to reduce ammonia absorption into the blood, thus playing a role in HE treatment [81]. The decrease of pH value is not conducive to the survival of urease-producing intestinal bacteria, but promotes the growth of nonurease-producing lactobacillus, resulting in the reduction of ammonia production in the colon lumen [82]. The incidence of OHE was lower in patients who received probiotics compared with those who did not [83].

Prebiotics are defined as ‘a substrate that is selectively utilized by host microorganisms conferring a health benefit’. Prebiotics are typically poorly digested fibrous compounds that feed beneficial bacteria in the digestive system, thereby regulating the host microbiome [84]. Prebiotics work by encouraging gut microbes to produce beneficial metabolites. Such as SCFAs, which can benefit the intestinal barrier and thus prevent intestinal permeability from increasing. Other beneficial effects of prebiotics include boosting ion and micronutrient absorption and enhancing host immunity [85].

Antibiotics for the treatment of HE rely on improving the intestinal environment by reducing the number of potentially pathogenic bacteria. It usually reduces the number of urease producing microbes. Some antibiotics selectively inhibit potentially pathogenic bacteria while allowing beneficial bacteria to survive. Due to the limitation of drug side effects and resistance, rifaximin is the antibiotic that has a clear role in HE [86]. Rifaximin is an oral drug with poor absorption and broad antibacterial activity. It had an apparent beneficial effect on the improvement of OHE, reversal of MHE, and prevention of recurrent HE [87]. After treatment with rifaximin, patients’ cognition improved significantly, endotoxemia decreased, and serum long-chain fatty acids increased significantly. It is suggested that the mechanism leading to cognitive improvement is not only related to changing the number of beneficial or harmful bacteria but also may be related to changing the metabolic function related to the gut microbiota [88].

## Future directions

The human gut is home to trillions of microbes, many of them in the gut. These microbes have been shown to affect the normal physiological functioning of body systems. Disruption of the microbiome balance in the gut has been contacted with some disease states, including gastrointestinal disease, neurological disease, cardiovascular disease, and even cancer. Therefore, targeted regulation of these microorganisms to promote overall health and eliminate disease is a new hope.

Microbiome-targeted therapy for HE has been studied and makes great progress, especially in FMT. However, the routine use of antibiotics to treat HE and spontaneous peritonitis in cirrhosis can disrupt the intestinal flora, so drug therapy combined with reasonable intestinal intervention is a double guarantee. Due to the complexity of cirrhosis and HE, existing studies on FMT need to expand the sample size to further determine factors, such as its safety, sustainable duration, duration of administration, optimal characteristics of donor feces, and so on. Donor selection is very important. In one study, two patients who were treated with FMT from the same donor developed extended-spectrum beta-lactamase (ESBL)-producing *Escherichia coli* bacteremia [89]. One of the patients died. Therefore, donor screening must be strengthened to reduce the transmission of microorganisms that may lead to adverse infection events. In addition to SCFAs and bile acids, other unknown factors await exploration for the beneficial bacterial products produced by regulating intestinal microbiota.

## Patient and Public Involvement

Patients or the public were not involved in the design, conduct, reporting, or dissemination plans of our research.

## Abbreviations

ACh: acetylcholine
APC: antigen-presenting cell
ARG: antibiotic resistance gene
BBB: blood–brain barrier
BDNF: brain-derived neurotrophic factor
CCl4: carbon tetrachloride
CDCA: chenodeoxycholic acid
CHE: covert HE
CNS: central nervous system
DCA: deoxycholic acid
ENS: enteric nervous system
FMT: fecal microbiota transplantation
GF: germ-free
HE: hepatic encephalopathy
ICT: inhibitory controlled trial
LPS: lipopolysaccharide
mAChR: muscarinic acetylcholine receptor
MHE: minimal HE
NG: nodose ganglion
OHE: overt HE
pTreg: peripheral regulatory T
SCFA: short-chain fatty acid
TCA: tricarboxylic acid cycle
TNF: tumor necrosis factor
VGx: subdiaphragmatic vagotomy
VN: vagus nerve
